# Sexual Dimorphism in Aggression: Sex-Specific Fighting Strategies Across Species

**DOI:** 10.3389/fnbeh.2021.659615

**Published:** 2021-06-28

**Authors:** Matias Pandolfi, Maria Florencia Scaia, Maria Paz Fernandez

**Affiliations:** ^1^Department of Biodiversity and Experimental Biology, University of Buenos Aires, Buenos Aires, Argentina; ^2^Department of Neuroscience and Behavior, Barnard College of Columbia University, New York, NY, United States

**Keywords:** aggression, invertebrates, model system, sexual dimorphism, territoriality

## Abstract

Aggressive behavior is thought to have evolved as a strategy for gaining access to resources such as territory, food, and potential mates. Across species, secondary sexual characteristics such as competitive aggression and territoriality are considered male-specific behaviors. However, although female–female aggression is often a behavior that is displayed almost exclusively to protect the offspring, multiple examples of female–female competitive aggression have been reported in both invertebrate and vertebrate species. Moreover, cases of intersexual aggression have been observed in a variety of species. Genetically tractable model systems such as mice, zebrafish, and fruit flies have proven extremely valuable for studying the underlying neuronal circuitry and the genetic architecture of aggressive behavior under laboratory conditions. However, most studies lack ethological or ecological perspectives and the behavioral patterns available are limited. The goal of this review is to discuss each of these forms of aggression, male intrasexual aggression, intersexual aggression and female intrasexual aggression in the context of the most common genetic animal models and discuss examples of these behaviors in other species.

## Introduction

Aggression is a complex, plastic behavior whose manifestation depends on an animal’s internal physiological state, sensory stimuli, and previous social experiences. Agonistic behavior is a more broadly defined concept, an adaptive act that arises from a conflict between two members of the same species. These behaviors play roles in conflict resolution when animals compete for specific resources such as territory, mates, or food sources and may involve intimidation of conspecifics by threat displays and can result in submissive responses like freezing, passive coping, or escape. Dominance in animals is established through repeated agonistic interactions that result in one animal controlling a contested resource. In animals living in groups, individuals who win agonistic encounters will become dominant, and losers often become subordinated, ultimately generating a hierarchical social organization. Some species establish a social hierarchy during the reproductive season that grants increased access to resources and reproduction to the highest-ranked individuals (Dewsbury, 1982). In social animals, at least three different types of social conflicts can be observed: between dominant and subordinates (Clement et al., 2005), among subordinates (Alonso et al., 2012), and between territorial neighbors (Muller and Manser, 2007).

Across species, competitive aggression is more common among males than females, and these differences are typically attributed to the action of steroid hormones during development or in adulthood (Gatewood et al., 2006). This is also the case in the best-studied rodent models in laboratory settings, including mice, rats, prairie voles, and hamsters. However, in all of these species, both males and females exhibit patterns of agonistic behavior, although often in different contexts (Been et al., 2019). Although female–female aggression is usually considered a behavior displayed almost exclusively to protect offspring, there are multiple instances of competitive aggression among females, both over high-value mates and food sources, in invertebrate and vertebrate species. Moreover, cases of intersexual aggression have been observed in a variety of species, particularly in fish.

Aggressive behavior studies in the laboratory have shed light on the genetic and neural basis of this behavior in animals that range from crustaceans to primates (Nelson, 2006; Anderson, 2012; Asahina, 2017). In particular, genetically tractable models have proven extremely valuable for our understanding of the underlying neuronal circuitry and the genetic architecture of aggressive behavior. Of all genetically tractable model organisms, the mouse nervous system is the most similar to ours. Studies in mice have shed light on mechanisms that appear to also regulate aggression in humans and may provide insight into psychiatric disorders associated with pathological aggression. Research in invertebrate models, *Drosophila* in particular, has also contributed to our understanding of neuronal mechanisms underlying this highly conserved behavior (Kravitz and Fernandez, 2015; Asahina, 2017). However, studies in genetic model systems also have limitations, particularly the lack of an ethological perspective. In this review, we compare the most commonly used genetic model systems, mice, zebrafish, and *Drosophila*, and discuss their advantages and limitations for studying three different forms of aggressive behavior: male intrasexual aggression, intersexual aggression, and female intrasexual aggression. In each case, we also discuss behavioral repertoires from other rodent, fish, and invertebrate species, that make them interesting and valuable models for the study of aggressive behavior.

## Male Aggression: Models for Competition and Territoriality

Mice are currently the most common laboratory animal model for the study of aggressive behavior. Aggression in mice is almost exclusively observed among males, and the most common test is the resident-intruder test, in which a resident animal confronts an intruder. This test allows the manifestation of both offensive and defensive behaviors. Early in the study of the neurobiological basis of aggression, rats, and hamsters were used for lesion experiments because they were the organisms most commonly used in behavioral psychology (Huhman, 2006). Those studies proved particularly insightful for our understanding of the brain regions that control male aggressive behavior, particularly the hypothalamic area, which has been linked to aggression for almost a century (Kruk, 1991). Studies in rats revealed that electrical stimulation of the so-called hypothalamic attack area (HAA) induces escalated aggression, which can be directed toward both males or females, or even mice (Kruk, 1991; Hrabovszky et al., 2005). However, in these approaches the spatial resolution for localizing specific neuronal populations involved in aggression is quite limited (Anderson, 2012). In contrast, the genetic tractability of mice has allowed an increasingly detailed mapping of neural circuits underlying aggression (Takahashi and Miczek, 2014).

Genetic tools available in mice, such as optogenetics, have also made it possible to explore the connection between areas related to aggression and other brain centers, and the tools for manipulation of neuronal activity and addressing neuronal connectivity in this species have enabled the identification of a specific population within the hypothalamus as crucial for male aggressive behavior. Studies using channelrodopsin2 showed that optogenetic stimulation of neurons in the ventrolateral subdivision (VMHvl) of the ventromedial hypothalamus (VMH) elicits aggression in male mice toward other males but also females and inanimate objects (Lin et al., 2011). Interestingly, this structure also contains neurons that appear to be active during mating (Lin et al., 2011). Silencing of the same neuronal population via an ivermectin (IV)-gated chloride channel led to a significant reduction in aggression, and in some cases its complete suppression, without affecting male mating behavior. Genetic tools have also allowed to study the connections between neuronal circuits that control aggression and brain areas that regulate other behaviors. For example, a recent study showed that circadian regulation of male aggression is mediated by a polysynaptic pathway from the suprachiasmatic nuclei to VMHvl neurons (Todd et al., 2018).

Although the mouse has many advantages as a model system, particularly the comparative ease of genetic manipulations and the possibility of circuit mapping, it also has limitations, notably the lack of extensive information about behavior in natural environments. However, experimental methods using environments more similar to the natural burrow system of the ancestral species have been developed to address some of the shortcomings of the standard behavioral tests and allow more ethologically relevant studies of dominance relationships in large groups (Williamson et al., 2017, 2019). Work from the Curley laboratory showed that groups of 12 outbred males mice living in large and complex environments establish linear and stable social dominance hierarchies (So et al., 2015; Williamson et al., 2016). Authors were able to subdivide the animals into three broad social status categories. Once a hierarchy is established each animal displays agonistic or subordinate behaviors to other males depending on the other animal’s relative social rank (Lee et al., 2018). Subordinate individuals are less likely to initiate fights than alpha or subdominant mice and lose far more contests than they win. Alpha males, which have the highest social rank, rarely lose fights and initiate a large fraction of agonistic interactions. Besides the behavioral consequences of establishing dominance these males also show higher levels of major urinary proteins and increased their feeding and drinking levels (Lee et al., 2017, 2018; Williamson et al., 2017). In addition, studies of large groups in complex environments revealed that socially dominant males had significantly higher oxytocin receptor (OTR) binding in the nucleus accumbens core than subordinate animals. Alpha males also showed higher OTR binding in several brain regions, while alpha males had lower vasopressin 1a receptor (V1aR) compared to subordinates (Lee et al., 2019).

Finally, the sensitivity of neural circuits mediating aggression and the behavioral responses to the effects of steroid hormones can vary greatly across species (Romeo et al., 2003). In mice, the direct connection between elevated testosterone and increased aggression is clear, whereas in other species such as Syrian hamsters (*Phodopus sungorus*), there relationship is reversed: testosterone decreases aggression in adult males housed under short-day conditions (Jasnow et al., 2000). Conversely, males from other rodent species, such as Mongolian gerbils (*Meriones unguiculatus*) and prairie voles (*Microtus ochrogaster*), do not exhibit diminished aggression in response to castration (Christenson et al., 1973; Demas et al., 1999).

### Aggression in Teleosts

In vertebrates, most social behaviors are regulated by the Social Decision-Making Network (SDMN), an evolutionarily conserved brain network in which consensus homologies for most relevant brain areas have been already identified in mammals, birds/reptiles, amphibians, and fish (O’Connell and Hofmann, 2011, 2012). Considering that the neural substrate for social behaviors is phylogenetically conserved and that fish species present a vast repertoire of reproductive and parental care behaviors, teleost fish constitute a group of growing interest in the study of aggressive behavior. In particular, the zebrafish (*Danio rerio*), native to freshwater habitats in South Asia, has become a widely used vertebrate model organism. Some of the advantages of zebrafish for aggression studies are their relatively small size, short generation time, and early onset of displays of social behaviors (Dreosti et al., 2015). Due to its fully sequenced genome, a wide offer of genetic tools including mutant lines, CRISPR/Cas9 genome engineering (Prykhozhij et al., 2017), and optogenetics (Del Bene and Wyart, 2012) has been established, as well as robust behavioral tests (Norton and Bally-Cuif, 2010). In recent years, zebrafish has been used to investigate several aspects of aggressive behavior, including the search for novel drugs that modulate aggressive behavior (Gutierrez et al., 2020).

Zebrafish male–male aggression is assessed during dyadic fights, after which a winner and a loser emerge as a consequence of a clear asymmetry of expressed behaviors, such as displays, circles, bites, chases, strikes, flees, and freezing (Oliveira et al., 2011; Teles and Oliveira, 2016). The temporal organization of these behavioral patterns allowed identifying highly structured patterns of aggressive behaviors. Not only contributing genes but also underlying neuronal pathways have been identified in this species, such as the hypothalamo-neurohypophysial or hypothalamo-pituitary-gonadal systems, and the histamine pathway, novel for non-mammalian systems (Filby et al., 2010). Interestingly, two subregions of the dorsal habenula antagonistically regulate the outcome of social conflict in zebrafish. While silencing the lateral subregion of dorsal habenula causes a stronger predisposition to lose a fight, silencing the medial subregion of the dorsal habenula is linked to winning the encounter, suggesting that both subregions of the habenula and their projections to the interpeduncular nucleus constitute a dual control system for conflict resolution (Chou et al., 2016). Remarkably, 69% of zebrafish genes have human orthologs. One of the limitations of zebrafish is that very little information is available about its behavior in natural environments. Therefore, while it is a useful model for studying the neural circuits that modulate aggressive behavior, currently studies in this model do not include an ecological or evolutionary perspective.

An attractive fish model to study aggression is the Siamese fighting fish *Betta splendens*. Popular in the aquarium trade, in the wild this species is found in standing waters of canals, rice paddies, and floodplains (Mendez-Sanchez and Burggren, 2014). Most of the animals used in research came from laboratory-reared animals, which are larger, more colorful, and substantially more aggressive than their wild counterparts, occasionally exhibiting lethal aggression between males in laboratory conditions. The differences between wild and laboratory-reared fish are quantitative and also involve divergent behavioral patterns (Ramos and Goncalves, 2019). The neural circuits and brain areas involved in aggressive behaviors in *B. splendens* have been studied primarily in strains that were artificially selected. *B. splendens* exhibit robust and highly stereotyped displays of aggressive behavior. Although it is not a genetically tractable model, CRISPR/Cas9 gene editing can be successfully implemented in this species (Andres Bendesky, personal communication). Gene editing techniques are also been implemented in other fish species (Gratacap et al., 2019; Warren et al., 2021).

From an ethological perspective, Cichlid fish are a particularly interesting model. The African cichlids, *Oreochromis mossambicus*, which is found on the Limpopo and Zambezi Rivers, and *Astatotilapia burtoni*, from Lake Tanganyika [reviewed by Fernald and Maruska (2012)], are the most extensively studied species to date. Cichlids form hierarchical social systems in which dominant individuals defend their status by aggressive displays toward other submissive, lower-ranked animals (Maruska, 2014). In particular, *A. burtoni* is a maternal mouth-brooding species living in a lek like social system, in which males can adopt two distinct reversible phenotypes: while dominant males are brightly colored and represent only 10–30% of the population, subordinate males present faded coloration and make up the majority of male population (Maruska and Fernald, 2013). Dominant males defend territories providing food, shelter, and substrate for spawning. While subordinates do not hold territories, they typically do not reproduce but school with females and other subordinates.

Since suitable territories are often limited, and females are less prone to mate outside shelters, *A. burtoni* males often engage in high-intensity aggressive encounters. They can reversibly switch between dominant and subordinate states, which has profound effects on behavioral and physiological mechanisms regulating reproduction. This fish model offers several important advantages for the study of the physiological basis of aggression: as in *B. splendens*, social change in males is signaled by obvious color differences which occurs within a few minutes, this species offers relatively easy access to the brain, facilitating sampling, and also has a fully sequenced genome (Fernald, 2012; Maruska and Fernald, 2013). Moreover, their social system can be replicated and manipulated under laboratory conditions, for example, mimicking natural changes to identify key physiological mechanisms regulating aggressive behavior and their impact on reproduction.

As in many other species, aggression in *A. burtoni* appears to be inhibited by serotonin (5-HT). Serotonin receptors in the telencephalon play a role in social status, and dominant males have more 5-HT cells in the raphe than their subordinate counterparts (Loveland et al., 2014). In addition, arginine vasotocin (AVT) regulates different aspects of male social behavior. While neuronal subpopulations in the parvocellular pathway are involved in the activation and modulation of submissive neural circuits or inhibition of aggressive/dominance networks, gigantocellular pathways have been associated with an upregulation of both courtship and aggression (Greenwood et al., 2008).

Steroid hormones have also been shown to play a role in aggressive behavior in *A. burtoni*. Growing evidence suggests that 17b-estradiol (E2) and brain aromatase, the enzyme that converts testosterone (T) to E2, have a central role in regulating male aggression. Dominant and territorial males also present higher circulating plasma levels of T, 11-ketotestosterone (11-KT, one of the most relevant androgens in fish), progesterone (P), and E2 when compared to subordinate males (Maruska and Fernald, 2013). When males are given a chance to raise their rank, T, 11-KT, E2, and P plasma levels increase due to changes in social status, suggesting that interactions occurring during the establishment of dominance modulate sex-steroid levels (Maruska et al., 2013). Both dominant and submissive males show differences in androgen and estrogen receptor mRNA levels in several brain regions within the SDMN (Maruska et al., 2013). Furthermore, since aromatase promotes aggression through actions in the preoptic area and estradiol promotes male aggression (Huffman et al., 2013), elevated T levels in dominant males can regulate aggression through their aromatization to E2 and a concomitant activation via estrogen receptors in the brain (Renn et al., 2008).

Although most of the research has focused on African cichlids, male aggressive behavior has also been studied in several Neotropical cichlid species. *Cichlasoma dimerus* (Chanchita) is an appealing model for studying the relations between hormones, social context, and behavior (Scaia et al., 2020). Unlike African cichlids, Chanchita is a monogamous species with biparental care, in which both males and females aggressively defend their territory (Pandolfi et al., 2009). This allows the study of aggressive parental behavior and underlying physiological mechanisms in both males and females. In this species, 5-HT also plays a key role in regulating male aggressive behavior. Evidence suggests that incorporating the rate-limiting substrate for 5-HT synthesis, the amino acid *L*-tryptophan, into the diet reduces the motivation to attack and modulates both aggressive and submissive behaviors (Morandini et al., 2019). After hierarchy establishment, subordinate males showed increased soma area of the parvocellular AVT subpopulation compared to territorial males, suggesting that changes in the synthesis or accumulation of AVT are necessary for the modulation of social behaviors (Ramallo et al., 2012). Steroid hormones also play a role in *C. dimerus*; while territorial, dominant males with high levels of aggression show higher T and 11-KT plasma levels than non-territorial males, the opposite is true for E2 (Ramallo et al., 2015).

### Invertebrate Models of Aggression

Invertebrates have proven to be excellent models for studying the neurobiological bases of aggression. Long before the introduction of *Drosophila*, the most widely used invertebrate genetic model, work on several invertebrate species, particularly crustaceans, revealed key aspects of the neural architecture underlying aggressive behavior, the formation and maintenance of dominance relationships, and the neurochemical mechanisms involved in the manifestation of aggression. These species typically have highly structured, accessible nervous systems, and aggressive behavior is highly stereotyped (Huber et al., 1997b; Kravitz and Huber, 2003). In crustaceans such as lobsters and crayfish, winners raise their legs and direct their antennae forward to display a dominant posture while losers adopt submissive postures (Huber et al., 1997b; Kravitz and Huber, 2003). Lobsters (*Homarus americanus*) became a model for the study of aggression largely due to their modular neural system, with few aminergic neurons (Kravitz and Huber, 2003). Amine neurons, in particular serotonin and octopamine, regulate their agonistic behavior, escalation of fights, and establishment of dominance (Kravitz, 2000). Laboratory studies on lobster aggression have focused on male–male encounters, in which opponents can cause serious injuries to one another. Agonistic encounters involve highly stereotyped behavioral patterns, progressing through visual displays to physical attacks of increasing intensity. Males initiate agonistic encounters even in the absence of females or resources, and unlike social animals, they form strong dominance relationships purely based on physical superiority (Huber et al., 1997a).

One of the main advantages of *Drosophila melanogaster* as a model for the study of aggression is its unparalleled genetic tools, which allow for high spatial and temporal resolution in manipulations of gene expression and neuronal activity (Venken and Bellen, 2007; Bellen et al., 2010; Kravitz and Fernandez, 2015). In addition, its highly stereotyped patterns of aggressive behaviors are robust across laboratory settings and make quantification straightforward and suitable for automatic tracking methods (Dankert et al., 2009; Kravitz and Fernandez, 2015; Asahina, 2017; Chowdhury et al., 2021). As in most species, males exclusively attack other males. *D. melanogaster* males are territorial, and that they fight over resources such as females and food. After several encounters, dominance relationships are established, and animals that have lost fights are less likely to engage in aggressive interactions against naïve individuals or familiar winners (Penn et al., 2010; Trannoy et al., 2015, 2016). Selecting for highly aggressive lines over several generations allows the generation of hyper-aggressive lines, and the study of the genetic contributions to this behavioral phenotype (Dierick and Greenspan, 2006; Penn et al., 2010). In male–male encounters, hyper-aggressive animals show shorter latencies to fight and increased retaliation frequency, and win the vast majority of fights against males of the original parental line. In *D. melanogaster* as well as in other invertebrates, serotonin seems to increase, rather than decrease, aggression (Huber et al., 1997b; Dierick and Greenspan, 2007). Interestingly, studies in other species suggest that this inverse relationship between serotonin and aggression does not hold across all invertebrates (Stevenson et al., 2000; Bubak et al., 2020). The brain connectome is close to completion in *D. melanogaster* (Li et al., 2020; Scheffer et al., 2020). When combined with existing genetic, physiological, and behavioral methods, this new knowledge will undoubtedly improve our understanding of how neural circuits control complex and plastic behaviors like aggression.

Both male and female *Drosophila* show aggressive behavior toward individuals of their same sex, but the behavioral patterns employed are highly dimorphic (Dow and von Schilcher, 1975; Jacobs, 1978; Nilsen et al., 2004). Moreover, only males establish dominance (Nilsen et al., 2004). The latency to start a fight is usually defined as the latency to the first *lunge*. Lunging is the most distinctive male pattern of aggression, a direct attack in which a male fly rises on its hind legs and snaps down on the opponent. Eventually, the dominant male gains control of the contested resources, after which the defeated animal retreats (Yurkovic et al., 2006; Miczek et al., 2007). In recent years, putative pheromones, as well as some olfactory and gustatory receptors, have been shown to play key roles in *Drosophila* aggression (Yew et al., 2009; Fernandez et al., 2010; Wang and Anderson, 2010; Wang et al., 2011), and the vast and versatile genetic toolkit available this species has made it possible to map neuronal circuits underlying this behavior (Asahina et al., 2014). However, as is the case for mice and zebrafish, studies in this species lack an ethological perspective.

The first descriptions of *Drosophila* aggression were those by Alfred Sturtevant in 1915, working mainly with *D. ampilophila* (Sturtevant, 1915). In an article about sex recognition and sexual selection, he was the first to mention male intrasexual aggression which appeared to be in the context of competition for mating partners. One of the patterns that he described for males appears to be similar to the “head-butt” pattern seen in *D. melanogaster* female fights (Nilsen et al., 2004). A few decades later A. Hoffmann used *D. melanogaster* and *D. simulans* and created a complete ethogram of agonistic interactions between males of the two species. Escalation of fights in *D. simulans* was more frequent and depended on body weight differences, and encounters lasted longer. More *D. simulans* males exhibited territorial behaviors (Hoffmann, 1987a,b).

A particularly interesting aggression phenotype that highlights the role of ethologically relevant environments has been described in males of the Mediterranean field cricket *Gryllus bimaculatus*. Similar to male lobsters, fights between male crickets follow a stereotyped sequence of escalating intensity. Initial encounters involve antennae, then proceed to spread mandibles displays, then interlocking mandibles and eventually engaging in “wrestling.” Losers tend to avoid further aggressive encounters. Remarkably, being allowed to fly after losing a fight restores their willingness to engage in subsequent fights, since losers regain their aggressiveness after being repeatedly thrown into the air (Hofmann and Stevenson, 2000). The majority of the losers re-engage in aggressive interactions with their previous opponent, and can escalate to the same level as naive animals. This is a rare example of activation of a motor pattern immediately after an aggressive interaction affecting the dynamics of the fight. Amine neurons have been mapped in the *G. bimaculatus* nervous system, and depletion of biogenic amines affects male aggression: the aggressiveness of crickets is reduced after depleting octopamine and dopamine from the CNS but is unaffected by serotonin depletion (Stevenson et al., 2000), suggesting that amines used to control aggression play different roles in insects and crustaceans (Stevenson et al., 2000; Murakami and Itoh, 2001). Moreover, the frequency and intensity of fighting can vary markedly within cricket species (Sakaluk, 1987; Jang et al., 2008; Kim et al., 2011).

In contrast to *Drosophila*, male–male fights in lobsters, crayfish, and crickets involve high intensity patterns of aggression and may result in physical harm. Recently, CRISPR/Cas9 gene editing has begun to be used to manipulate gene expression in crustaceans (Martin et al., 2016; Xu et al., 2020). This opens the possibility of expanding the applications of some of the classical invertebrate models to study of the neurobiology of aggression by adding genetic tools that could enable, for example, optogenetic control of neuronal activity during fights in natural or semi-natural environments.

## Intersexual Displays of Aggression

In mice, female aggression toward males is rare and has been described mostly in the context of maternal aggression. Female aggressive behavior is frequent before gestation, increases shortly postpartum, and then declines (Noirot et al., 1975; Erskine et al., 1978). Maternal aggression includes both defensive and offensive behavioral patterns. Lactating females engage in defensive attacks toward males and offensive attacks toward female intruders (Lucion and de Almeida, 1996). Unlike male–male aggression, which has been widely studied in species ranging from invertebrates to primates, little is known about mechanisms underlying male attacks toward conspecific females. Males from the most widely used mammalian genetic model, mice, do not normally attack females under laboratory conditions. However, optogenetic activation of the VMH elicits male aggression toward females and toward inanimate objects (Lin et al., 2011).

In contrast, pair-bonded prairie voles exhibit one of the most robust aggressive responses from a male rodent toward a female. Specifically, once a pair bond has been formed, males exhibit aggression toward conspecific females but not toward their partners. This response appears to be mediated by dopamine receptor expression in the nucleus accumbens (Young and Wang, 2004). Vasopressin also plays a role, and the anterior hypothalamus (AH)-AVP system appears to mediate aggression toward females in hamsters as well as in other rodents (Ferris et al., 1989; Motta et al., 2009). Interestingly, cohabitation with females in the absence of mating does not induce male attacks toward novel females (Insel et al., 1995; Wang et al., 1997). Males that mated for 24 h and formed bonds attacked both male and female intruders and expressed higher levels of Fos-ir expression in the medial amygdala (meA) (Wang et al., 1997). Selective aggression appears to serve the role of maintaining the monogamous pair bond (Resendez and Aragona, 2013), and is a rare example of a case in which rodent males display aggression toward a sexually receptive female that does not represent a threat. Thus, the prairie vole is considered an outstanding model for studying the neuronal mechanisms underlying monogamy in rodents.

### Intersexual Aggression in Teleosts

Intersexual aggression in zebrafish has been assessed mainly in the context of how size-selective harvesting (e.g., fisheries) can directionally change sexually selected traits. To study the role of the size-selective harvesting on the evolution of mating behavior, size-matched spawning trials were performed among different size-harvested lines of zebrafish (Sbragaglia et al., 2019). Evidence suggests that while male aggression is lower when random-harvested males were crossed with females from the small and random harvested lines, male aggression is higher when large and small harvested males were crossed with females from the random harvested line. Moreover, females from the large harvested line experience lower levels of male aggression than females from the random and small harvested lines. Interestingly, since evidence on intersexual aggression in zebrafish focuses on male aggression because of its key importance in mating behavior of this species (Spence et al., 2008), female aggression toward males is still understudied.

*Betta splendens* males and females intensely and frequently attack each other regardless of the reproductive context and males often attack females for long periods of time. Females also attack males, but less frequently. In contrast, intersexual aggression in cichlids is often associated with pair-bonding and reproductive behavior. The convict cichlid (*Amatitlania siquia*) is a serially monogamous fish in which both intrasexual competition and intersexual selection influence the mating pattern. In this species, both sexes are highly aggressive, and the winner of aggressive encounters is usually the larger individual regardless of sex (Leese, 2012). Several monogamous species demonstrate size-assortative mating patterns, showing a positive correlation between male and female sizes of mate pairs within a population. In the case of the convict cichlids, oftentimes pairs are formed in which males are larger than females both under laboratory (Beeching and Hopp, 1999) and field conditions (Wisenden, 1994). Intersexual selection in this species influences size-assortative mating, and most studies have focused on female preference for larger males (Gagliardi-Seeley et al., 2009). However, when males are forced to pair with a smaller or a larger female, pair formation only occurs when the female is smaller than the male, while larger female shows high aggression to the male (Bloch et al., 2016). This suggests that intersexual aggression from females toward males limits size-assortative mating. Moreover, there is also evidence of intersexual aggression from males toward novel females (Leese, 2012). Besides cichlids, another interesting organism for the study of intersexual aggression is the electric fish *Gymnotus omarorum*, which shows non-breeding intrasexual and intersexual territorial aggression, does not exhibit sexual dimorphism in body size and in which body size and not sex is the best predictor of dominance in intersexual aggressive encounters (Batista et al., 2012).

### Intersexual Aggression in *Drosophila*

Intersexual aggression in *D. melanogaster* has not been observed in the absence of manipulations of neuronal activity or gene expression, at least under laboratory conditions. High levels of female aggression toward males can be elicited by masculinization of the female nervous system either via expression of the male form of *fruitless* (Vrontou et al., 2006) or through RNA interference (RNAi)-mediated silencing of the sex determination gene *transformer* (Chan and Kravitz, 2007). Such “masculinized” females attack females of the same genotypes, mutant, or transgenic males that exhibit female aggression patterns, and wild-type males. However, these manipulations do not trigger female aggression toward wild-type females.

*Drosophila* males attack only other males and do not attack females (Kravitz and Fernandez, 2015). However, when the pheromonal profile of females is genetically masculinized, these females can elicit aggression from males (Fernandez et al., 2010). The composition of female cuticular hydrocarbons (CHs), which serve as contact pheromones, can be changed to that normally found on surfaces of the males by expression of a *transformer* RNAi transgene (Fernandez et al., 2010). These females exhibit pheromonal profiles similar to those of wild-type males, with high levels of monoenes and low levels of dienes. Males also attack females with a masculinized central nervous system, indicating that behavioral cues displayed by the females can override their chemical cues (Fernandez et al., 2010). In addition, males in which the nervous system is feminized by expression the female form of *fruitless* (Vrontou et al., 2006) or in which *transformer* is ectopically expressed (Chan and Kravitz, 2007) exhibit aggression toward females. Interestingly, activation of tachykinin-expressing neurons in males can elicit male aggression toward females (Asahina et al., 2014).

## Female–Female Aggression

Although not as extensively studied as male intrasexual aggression, female intrasexual aggression occurs in vertebrate and invertebrate species (Clutton-Brock, 2009). Exploring the dynamics underlying this behavior in taxa with different evolutionary histories would help better understand the selective pressures driving the evolution of female aggression. Female–female aggression has been postulated to be the by-product of genetic correlations with males (Lande, 1980). According to this hypothesis, traits that are advantageous for males, like aggression toward other males, are often expressed in females as well (Forstmeier et al., 2011). An alternative explanation is that female–female aggression has evolved from direct selection on females themselves and likely functions in competition over reproductive and social benefits (Tobias et al., 2012; Stockley and Campbell, 2013).

One reason why studies of aggression have focused on males is the potentially confounding behavioral effects of steroid hormone level oscillations during the estrus cycle. Estradiol, the main steroid hormone in females, has been implicated in female aggressive behavior (Rosvall et al., 2012), and several rodent species such as rats and hamsters are less likely to exhibit aggression during the estrus cycle (Wise, 1974; Davis and Marler, 2004). However, in these species, as well as in mice, the effect of the estrus cycle on aggression remains unclear. As in males, brain regions in the social behavior network [meA, bed nucleus of the stria terminalis (BNST), lateral septum (LS), medial preoptic area (mPOA), AH, VMH, and periaqueductal gray (PAG)] as well as the mesocorticolimbic dopamine pathway have been found to form the basis of the neural circuit regulating aggression, though there are some important sex differences (Duque-Wilckens and Trainor, 2017).

Intrasexual competition and hierarchy formation in female mice appears to be rare when population density is low and increase as population size increases, but little is known about the formation of female social hierarchies in mice (Yasukawa et al., 1985; Weidt et al., 2018). As it is the case for males, studies in more naturalistic environments revealed novel aspects of female social behaviors that were not observed under standard laboratory conditions (Williamson et al., 2019). Female mice living in large, complex environments are able to form linear hierarchies that emerge quickly, are stable for around 2 weeks and do not appear to be affected by the estrous cycle. Interestingly, these females housed under these conditions showed an extended estrous cycle (Williamson et al., 2019). Dominant females spent significantly longer in estrus than subordinate females, which subordinate showed higher levels of plasma corticosterone than dominant females, suggesting that they may be more susceptible to social stress.

Unlike most female laboratory rodents, which rarely display spontaneous aggression, female Syrian hamsters exhibit a range of competitive strategies. Females are able to form robust and stable hierarchal relationships and even inhibit the reproductive capacity of other females (Albers et al., 2002). Interestingly, clear sex differences in the neural regulation of dominance and aggression have been reported in this species. While hypothalamic injection of a 5-HT1a agonist stimulated aggression in females and inhibited aggression in males, injection of AVP had the opposite effects on both males and females. In addition, formation of female dominance was associated with activation of 5-HT neurons within the dorsal raphe while formation of male dominance was associated with activation of AVP neurons in the hypothalamus. Interestingly, fluoxetine increased female aggression while it substantially reduced aggression in males, an observation with obvious implications for psychiatry (Terranova et al., 2016).

### Teleosts and Female Dominance

Analyses of aggression in fish have focused on male intrasexual competition. However, female dominance behaviors can also be observed in common laboratory models and in domestic fish. In zebrafish, although female intrasexual encounters are less aggressive (i.e., fewer attacks in the same time interval), evidence suggests that dominant females display significantly more aggressive displays than subordinate females (Filby et al., 2010). During the spawning period, dominant females are less aggressive toward their subordinate same-sex counterparts than dominant males toward theirs (Paull et al., 2010). Given that zebrafish is a popular vertebrate model for studying the neuronal basis of behavior, female aggression is surprisingly understudied. For example, a systematic quantification of aggressive behavior patterns is not yet available, and the role of all brain activation across the SDMN in aggressive displays remains unknown.

*Betta splendens* females exhibit a fighting pattern similar to that of males when in small aquariums (Braddock and Braddock, 1955). Fights between females end with submissive behaviors displayed by one of the individuals, while the dominant female continues to exhibit aggression for a short period of time. When housed in mixed large groups, female–female fights are less frequent than male–male fights (Elcoro et al., 2008). Aggression in female wild-types has also been described and compared to a strain that was selected for more aggression (“fighters”) by replicating a mating scheme commonly used by local breeders in Thailand, in which sibling males of a winner are mated with sibling females from another breeder (Ramos and Goncalves, 2019). ‘Fighter’ females are more aggressive than wild-type females, but the differences are quantitative rather than qualitative. Even though both strains show similar behavioral patterns (frontal displays, lateral displays, charge, caudal swing, and approach), behavioral correlation networks of the two strains are similar when females are paired with conspecifics but different in the mirror trials. Higher aggression in fighter females may be an adaptation to captivity, with more aggressive females having higher survival rates.

There are several examples of species in which females display high levels of aggressive behavior. Fish present a wide variety of reproductive and parental strategies, and cichlid fish are particularly interesting models to study both male and female aggression. For example, both intrasexual male and female aggression has been reported in dyadic agonistic encounters in the cooperatively breeding cichlid *Neolamprologus pulcher* (Taves et al., 2009), and aggression levels are similar between the sexes. Newly dominant females have higher plasma testosterone (T) but similar 11-KT levels in comparison with newly subordinate females (Taves et al., 2009). By contrast, newly dominant males have higher 11-KT but similar T levels relative to subordinate males. Female aggressive behavior of an intensity comparable to that in males has also been reported in the cichlid *A. siquia* (Bloch et al., 2016).

In contrast, females of the cichlid *A. burtoni* are usually not aggressive and do not form social hierarchies. However, when they are placed in all-female communities, they develop social hierarchies, display aggression, and exhibit male-like patterns of behavior (Renn et al., 2012; O’Connell et al., 2013). Interestingly, in a recently collected stock of fish, females of this species show aggressive behaviors toward male intruders if they are taking care of their brood (Renn et al., 2009). When comparing the neuroendocrine regulation of aggression in male and female dominants and subordinates, there are sex-specific and status-specific patterns of hormonal regulation of dominance (Renn et al., 2012; O’Connell et al., 2013). Moreover, evidence on neural gene expression suggests that there are specific modules and functional gene ontology categories that can explain either dominance or reproductive state when comparing brooding females with dominant and subordinate males (Renn et al., 2008). In females, gene expression patterns reveal a core module of genes associated with social dominance and up-regulation of genes previously identified as male-biased (Renn et al., 2016). However, even if aggressive behavior in *A. burtoni* females is observed in recently collected fish or in tanks in only in all-female groups, these behaviors have been studied in the context of maternal aggression and not in neutral aquaria. Monogamous cichlids, as well as species without lek-like system, are interesting models for the study of female territoriality and the underlying neuroendocrine mechanisms (Reddon et al., 2013).

Females of the neotropical cichlid *C. dimerus* can be as aggressive as males, as dominant, reproductive females aggressively defend their territory from subordinate, lower-ranked animals (Ramallo et al., 2014). The highest aggression levels in pre-spawning females are associated with larger GnRH-3 nuclear and somatic area and peaks in androgen and E2 plasma levels (Tubert et al., 2012). Comparisons of male–male and female–female encounters in neutral arenas do not reveal significant differences between sexes in terms of latency to attack, time of resolution, or frequency of aggressive displays, suggesting that females are as aggressive as males (Scaia et al., 2018b). Moreover, female winners show higher E2 levels before the agonistic encounter than female losers, while there are no differences on T and 11-KT levels (Scaia et al., 2018a). These results suggest that in *C. dimerus* female aggression is associated with initial levels of E2, and that estrogen levels could predict female aggression.

### Female Aggression in Invertebrate Models

Aggression in *D. melanogaster* females was first described by Ueda and Kidokoro (2002). The authors described the female behavior as being similar to those of males identified and identified several behavioral patterns, including “lunge.” The “lunge” described in this study was different from the pattern used currently to quantify male aggression (Nilsen et al., 2004), since the female lunge did not involve rising. Ueda and Kidokoro reported that female aggression levels were dependent on rearing conditions, since isolated females were more aggressive than their group-housed counterparts, and on the quality of the food source, which suggested defense of potential future egg-laying sites.

Recent studies showed that female aggression in *D. melanogaster* is influenced by mating via an associated seminal fluid protein called sex peptide (Bath et al., 2017). Although the majority of the work on *D. melanogaster* aggression has been done in males, a growing number of studies have focused on female intrasexual aggression. Neuronal populations that mediate female-to-female aggression have been identified (Palavicino-Maggio et al., 2019), such as the *doublesex*-expressing pC1 cluster (Deutsch et al., 2020). Optogenetic activation of a subset of the neurons derived from the aIP-g neuroblast (Cachero et al., 2010) increases female aggression in the absence of aggression-promoting cues (Schretter et al., 2020). As is the case with males, connectomics studies in combination with genetic tools will likely help our understanding of how neuronal circuits control aggression with a level of resolution that is not possible in other invertebrate species. However, aggression in female *D. melanogaster* is less frequent and substantially less intense than in males, and females do not establish dominance (Nilsen et al., 2004). Studies focused on female competitive aggression in other non-social insects would allow for an examination of other aspects of this form of aggression. Unfortunately, there are relatively few such studies, as most focus on female aggression in social insects.

Female aggression in the context of nestmate recognition has been explored in multiple insect species such as ants, bees, wasps, and termites. One of the best described cases of female aggression in invertebrates is that of honey bees (Nouvian et al., 2016). Unlike other eusocial species, like ants and termites, guard, and soldier bees do not exhibit obvious morphological differences. Nest guards remain at the hive entrance to determine whether incoming individuals belong to the nest or are unfamiliar. Aggression is context-dependent and can be influenced by food availability: guards are less likely to attack non-nestmates when the colony has and guarding is decreased under high predation pressure [reviewed in Nouvian et al. (2016)]. The presence of a queen also affects honeybee defensive behavior: without a queen, all individuals participate in nest defense (Naeger et al., 2013). As in other invertebrates, central biogenic amines play a role in mediating aggressive behaviors: for example, octopamine decreases the activity of the stinger (Burrell and Smith, 1995). In addition, the genetic architecture of honeybee aggression has been well described (Hunt, 2007), and aggression-related changes in gene expression have been reported (Alaux et al., 2009).

## Concluding Remarks

Over the past two decades, genetic models such as *Drosophila* and mice have been extremely useful for understanding not only the role of genes but the neuroarchitecture underlying aggressive behavior. A sophisticated and increasingly versatile repertoire of genetic tools has enabled the identification of specific neuronal populations involved in aggression, and manipulation of gene expression and neuronal activity specifically in those neurons to elucidate their roles in aggressive interactions. Despite a recent increase in attention to females, the vast majority of the literature has focused exclusively in male–male interactions. Although these models, especially mice, have made unparalleled contributions to our understanding of the neurobiological mechanisms underlying aggressive behavior, each model has distinct limitations that are sometimes ignored. The ethological perspective is arguably the most important aspect missing from studies in genetic models. The frequency and patterns of behaviors manifested by animals under laboratory conditions often differ greatly from those displayed in the wild or even semi-natural environments. Technological advances will undoubtedly soon allow us to overcome some of the limitations, e.g., by adapting optogenetics manipulations to freely moving animals in large spaces.

More broadly, technology will also soon allow genetic manipulations in species that had been beyond experimental reach. CRISPR is already being used in a wide range of species, many of which are potentially valuable models for the study of aggression because they exhibit patterns of behaviors absent in mice or flies, such as lethal aggression or high-intensity patterns of female intrasexual aggression. These tools will expand the repertoire of organisms amenable to experimental manipulation and could help to bring back classical non-genetic models such as crustaceans, in which fights are highly stereotyped and have long-lasting consequences. In this new era, rather than being limited to the inquiries that can be made in a few laboratory models, researchers seeking a neuroethological perspective will be empowered to select their model organisms based on the biological question of interest.

## Author Contributions

MP and MPF conceived and wrote the manuscript. MFS wrote the manuscript. MFS and MPF gave their approval for final submission. All authors contributed to the article and approved the submitted version.

## Conflict of Interest

The authors declare that the research was conducted in the absence of any commercial or financial relationships that could be construed as a potential conflict of interest.
